# Ferroptosis and Neurodegeneration: Unlocking New Therapeutic Pathways in Alzheimer's Disease

**DOI:** 10.1002/alz70855_105436

**Published:** 2025-12-24

**Authors:** Ritika Saini

**Affiliations:** ^1^ MMCP, Ambala, Haryana, India

## Abstract

**Background:**

Alzheimer's disease (AD) is the most common cause of dementia, making up between 60% and 80% of cases. Even with a great deal of work over the years, the exact mechanism of AD has not been fully understood. The disease's pathophysiology is defined by neurofibrillary tangles and β‐amyloid plaque, however the mechanism underlying neurodegeneration remains unknown.

**Methods:**

Ferroptosis has been identified as a key player in the pathogenic process of AD in recent years. Neurofibrillary tangles (NFTs) and the deposition of senile plaques (SP) are both influenced by iron dyshomeostasis.

Ferroptosis is caused by reactive oxygen species (ROS) that come from a variety of sources, including the accumulation of free intracellular divalent iron (Fe2+), which causes polyunsaturated fatty acids (PUFAs) to peroxide and damages membranes.

Reduced intracellular availability of antioxidant enzymes, especially glutathione peroxidases (GPXs), is a key cause of ferroptosis. The neurological tissue is particularly susceptible to oxidative injury and ferroptosis due to its high lipid concentration and oxygen consumption rate. All nervous system cell types, including neurons, glial cells, and pericytes, are impacted by ferroptosis.

**Result:**

Iron dysregulation, particularly in microglia, contributes to the accumulation of reactive oxygen species (ROS) and oxidative stress, promoting neurodegeneration. The high lipid content and oxygen consumption in neural tissue make it particularly susceptible to ferroptosis. Understanding the pathways regulating ferroptosis in AD could offer insights for future therapeutic strategies.

**Conclusion:**

According to recent data, ferroptosis linked to neurodegeneration is significantly influenced by iron‐loaded microglia. In order to provide useful knowledge for the future treatment and prevention of AD, we examine here the primary pathways regulating ferroptosis in AD as well as the possible connection between AD and ferroptosis.

**Keyword**: Alzheimer, β‐amyloid plaque, ferroptosis, antioxidant, glutathione, neurodegeneration.

